# Using cluster edge counting to aggregate iterations of centroid-linkage clustering results and avoid large distance matrices

**DOI:** 10.14440/jbm.2017.153

**Published:** 2017-03-16

**Authors:** Matthew Kellom, Jason Raymond

**Affiliations:** School of Earth and Space Exploration, Arizona State University, Tempe, AZ 85287-6004 USA

**Keywords:** clustering, cluster, centroid-linkage, distance matrix, aggregate

## Abstract

Sequence clustering is a fundamental tool of molecular biology that is being challenged by increasing dataset sizes from high-throughput sequencing. The agglomerative algorithms that have been relied upon for their accuracy require the construction of computationally costly distance matrices which can overwhelm basic research personal computers. Alternative algorithms exist, such as centroid-linkage, to circumvent large memory requirements but their results are often input-order dependent. We present a method for bootstrapping the results of many centroid-linkage clustering iterations into an aggregate set of clusters, increasing cluster accuracy without a distance matrix. This method ranks cluster edges by conservation across iterations and reconstructs aggregate clusters from the resulting ranked edge list, pruning out low-frequency cluster edges that may have been a result of a specific sequence input order. Aggregating centroid-linkage clustering iterations can help researchers using basic research personal computers acquire more reliable clustering results without increasing memory resources.

## INTRODUCTION

Agglomerative clustering is a useful tool to bin sequencing datasets based on sequence similarity, but the increasing use of high-throughput sequencing technology is creating datasets large enough to make clustering impractical for some computers and/or clustering methods. The most basic and widely used sequence clustering techniques are agglomerative, creating hierarchical bins *via* joining algorithms such as minimum-, maximum-, and average-linkage, with average-linkage being the most popular due to its perceived accuracy [1-4]. One drawback to these methods is that they require the construction of exhaustive distance matrices containing relative difference information between all possible pairwise sequence comparisons. After a distance matrix is constructed, the average-linkage algorithm bins sequences into clusters if the mean distance between all cluster member sequences is at or above the chosen clustering cutoff level, with minimum- and maximum-linkage using alternative binning requirements.

**Figure 1.**
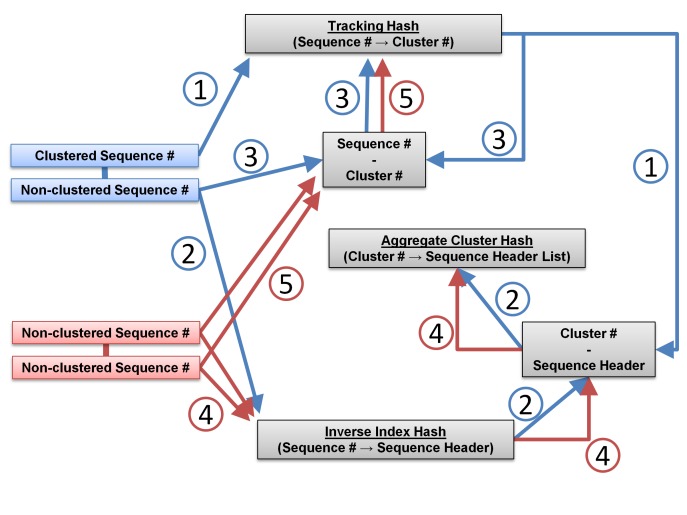
Flowchart of aggregating algorithm. Two scenarios are represented in the flowchart: One of the sequences for the current edge has already been assigned to a cluster from a previous edge according to the Tracking Hash (Blue); Neither sequence from the current edge has been assigned to a cluster according to the Tracking Hash (Red). A third scenario where both sequences of an edge have already been assigned to a cluster is not shown since that edge would be skipped in the algorithm. The processes in the flowchart have been numbered and described: (1) Using the Sequence Numerical Identifier of the already clustered sequence of the paired edge, obtain the Cluster Numerical Identifier from the Tracking Hash. (2) Using the Sequence Numerical Identifier of the non-clustered sequence of the paired edge, obtain its sequence header from the Inverse Index Hash and append it to the sequence header list value for the Aggregate Cluster Hash key of the Cluster Numerical Identifier from step 1. (3) Append the non-clustered Sequence Numerical Identifier and Cluster Numerical Identifier from step 1 to the Tracking Hash to finalize it as a clustered sequence. (4) Both Sequence Numerical Identifiers of the non-clustered pair are used to obtain their sequence headers from the Inverse Index Hash and assign them to a new Cluster Numerical Identifier key in the Aggregate Cluster Hash. (5) Both Sequence Numerical Identifiers are paired with their Cluster Numerical Identifier and appended to the Tracking Hash.

Distance matrix construction is a key computational bottleneck in agglomerative clustering. For large datasets, the computational needs of their distance matrices can exceed computer memory limits, especially for researchers using standard personal computers. Centroid-linkage clustering circumvents the need for a distance matrix at the cost of being input-order dependent, but this also makes the centroid-linkage algorithm faster and more memory-efficient for large-scale datasets than its agglomerative counterparts [5]. Since centroid-linkage clustering relies only on single pairwise sequence comparisons read in input file order, randomizing the order in which comparisons are made and centroids assigned can affect cluster-sequence distribution. A graphical example of how sequence input order can affect cluster-sequence distribution can be found in **Figure 1** of reference [6]. This means that depending on the sequence input order, a specific cluster edge between two sequences may or may not form, affecting sequence-cluster membership. To address this challenge, some have considered ordering input sequences by length or abundance, with some programs employing these techniques natively, like CD-HIT [7]. Sorting sequences by length ensures that cluster centroids contain maximum information and thus cluster members can be binned more accurately. Conversely, abundance sorting approaches accuracy with the assumption that abundant sequences are more likely to represent functionally relevant clusters. However, both of these sorting methods still produce results that are dependent on a single, and to some degree, arbitrary input order. This is discussed further in the Discussion section.

Standard clustering concepts still apply to centroid-linkage, more closely related sequences are more likely to form an edge and be assigned to the same cluster. Over enough iterations of input randomization and clustering, edges that represent closely matched sequences will appear in the majority of iterations. By keeping track of all of the edges and ordering them by most frequently formed throughout the iterations, we can essentially form an ordered list of the most closely related cluster edges. From this ordered list of cluster edges, we can piece back together the clusters and make sure that sequences end up binned in clusters where they have the most representative cluster edge. The purpose of this protocol is to provide biology researchers without access to sufficiently high-performance computing with a means to obtain sequence clustering results that do not require the construction of large distance matrices while also not being solely dependent on sequence input order. This process of random input order centroid-linkage clustering over multiple iterations, breaking down the resulting clusters into their individual edges, counting those edges, and then reconstructing aggregate clusters from a ranked edge list effectively bootstraps aggregate cluster edges from input-order dependent clusters and increases the reliability of centroid-linkage results.

This methodology is beneficial when the amount of available random-access memory (RAM) cannot contain the distance matrix being made, preventing agglomerative clustering processes from completing. For example, using traditional agglomerative clustering algorithms and a centroid-linkage algorithm in the program USEARCH (www.drive5.com/usearch/) allows for different limits on the maximum number of input sequences. Maximum-, minimum-, and average-linkage algorithms were only able to process ~10000 sequences past the distance matrix step on our 120 GB RAM-containing computer, capacity beyond what is typically thought of for a standard computer. By eliminating the need for a distance matrix, the number of sequences that the centroid-linkage algorithm is able to process is only limited by the size of the file that can be read into memory (> 1000000 for our 120 GB RAM computer). Importantly, these results do become input-order dependent. By avoiding distance matrices and writing edge lists and edge counts to text files in disk space (rather than storing in memory), the aggregation process is slower than agglomerative clustering but it is also more likely to finish before running out of necessary memory.

For comparison, the centroid-linkage algorithm was able to complete clustering of 10000 sequences in four seconds on our computer, while the minimum-, maximum-, and average-linkage algorithms each took eighteen seconds and the aggregation process took an hour and twenty-two minutes. As the number of sequences in a dataset increases, the runtime of the aggregating algorithm increases drastically (detailed in Results). The increased time is to be expected because not only is it waiting for multiple iterations of centroid-linkage clustering to complete, but it must also count and store all cluster edges. Although slower than average-linkage algorithms that use distance matrices for accuracy, this aggregation method is more likely to complete before running out of memory space. Likewise, as datasets and iterations increase, so does the amount of necessary disk storage. For our largest dataset of one million sequences over 101 clustering iterations, approximately 170 GB of data was written in the form of small individual text files. With this cost in speed and storage, aggregating multiple iterations of the efficient centroid-linkage algorithm increases the confidence of cluster-edge distribution for datasets that are too large to be clustered with comprehensive distance calculations.

## MATERIALS AND METHODS

The procedure outlined here includes the use of specific clustering and scripting programs but similar programs should work just as well. The choice of which programs is determined by user preference. The important details are to use a program that performs centroid-based clustering, or some other distance-matrix independent algorithm, and use a scripting language to perform the following aggregation procedure with the resulting clusters. The annotated Perl script used by the authors is supplied as **File S1**. Kolmogorov-Smirnov (KS) comparisons between different clustering methods and the aggregation process were performed in R with ks.test of the R Stats Package (r-project.org).

### Sequence indexing

Sequences are first given a numerical identifier (Sequence Numerical Identifier hereafter) by indexing the sequence order of the original input, avoiding potential downstream filename parsing errors. For the sake of speed, this index is stored in RAM as a hash table (Index Hash hereafter) with the sequence header as the key and the Sequence Numerical Identifier as the value (defined as hash{key} = value in Perl), but could be created and accessed in disk storage if desired. Typically, the amount of memory needed for this index is considerably smaller than what would be needed for a clustering distance matrix. It is very important during this indexing step for each of the input sequence headers to be unique so that later sequence header recall from their corresponding numerical identifiers can be done accurately. The sequences used to demonstrate the anticipated results originate from an unpublished metatranscriptome dataset with a mean sequence length of 98 bases and their origin is not important for the explanation of this methodology. Any natural dataset should yield similar clustering results to those seen in **Figure 1**.

### Clustering

Over sufficient iterations (the authors here chose 101 iterations), clustering is performed with the USEARCH (version 8.0.1517_i86linux64) “-cluster_fast” command at a 0.95 clustering threshold and clusters are written to separate files using the “-msaout” command (Cluster Files hereafter) [5]. The authors here chose 101 iterations (counting from 0 to 100) because the results were stable at this number. In general, more iterations will lead to more stable results, and larger datasets will need more iterations. Determining the appropriate number of iterations is specific to each individual case. The USEARCH “-cluster_fast” command utilizes centroid-based clustering and avoids creating computationally costly distance matrices at the cost of being input-order dependent. To mitigate the effects of input-order dependence, the sequence FASTA-formatted input file is first reordered randomly prior to clustering and downstream edge counting for each iteration. The Sequence Numerical Identifiers created in step 1 are not altered by the randomization process. Depending on the dataset, a smaller number of iterations may result in aggregate clusters that are dependent on those randomized clustering input files.

### Edge compiling

After clustering has completed for the chosen number of iterations, Cluster Files are accessed to begin counting edges. Singleton clusters containing only one sequence and no edges are ignored by the counting process, and this minimum edge parameter can be increased to speed up the compiling/counting process at the cost of comprehensiveness. Singletons and low-edge-count clusters are not typically represented in large aggregate clusters.

To avoid storing edge counts in RAM, which can quickly reach capacity for large datasets in typical research personal computers, edges are written to files in disk storage (Edge File hereafter) with the numerically lesser Sequence Numerical Identifier as the filename of the Edge File (Hub hereafter) and the higher Sequence Numerical Identifier as a line in the Edge File (Node hereafter) so that a specific edge’s count from the iterations can be obtained by counting the number of times a Node Sequence Numerical Identifier is found in an Edge File, this is important for the downstream edge counting.

### Edge counting

For each compiled Edge File, the counts of specific Nodes for each Hub are stored in new files with filenames that represent their count (Count File hereafter). This counts the number of times a specific edge appears by writing the Hub and Node on a single line, never exceeding the number of chosen iterations.

### Reconstruction

Aggregate clusters are reconstructed from the edges contained in Count Files, starting with the highest Count File (edges that were found the most in the iterations, typically equal to the number of iterations) and working down toward the lowest Count File. For the reconstruction algorithm, four hashes are created. First, the Index Hash created in step 1. Second, the inverse of the Index Hash, so that Sequence Numerical Identifiers are stored as keys and sequence headers as values (referred to as Inverse Index Hash in the algorithm below). Third, an aggregate cluster hash where keys are a numerical identifier assigned to clusters (Cluster Numerical Identifier hereafter) and values are lists of the sequence headers contained in each cluster (referred to as Aggregate Cluster Hash in the algorithm below). Fourth, a hash that tracks which Cluster Numerical Identifier (value) each hub and node are stored (key) (Tracking Hash in the algorithm below). For each edge of Hub and Node Sequence Numerical Identifiers, aggregate clusters are reconstructed using the following algorithm and then written to an output file:

1. Skip to the next edge if both the Hub and Node have already been assigned to clusters in the Tracking Hash.
2. If the Hub has already been assigned to a cluster in the Tracking Hash (implying with step 1 that the Node has not been assigned yet):
  2.1. Get the Cluster Numerical Identifier value that the Hub Numerical Identifier key has been assigned to in the Tracking Hash.
  2.2. Get the sequence header value for the Node Numerical Identifier key from the Inverse Index Hash and append it to the value for the Cluster Numerical Identifier (from step 2.1 key in the Aggregate Cluster Hash.
  2.3. Append this Node Numerical Identifier key - Cluster Numerical Identifier value pair to the Tracking Hash.
3. If the Node has already been assigned to a cluster in the Tracking Hash (implying with step 1 that the Hub has not been assigned yet):
  3.1. Get the Cluster Numerical Identifier value that the Node Numerical Identifier key has been assigned to in the Tracking Hash.
  3.2. Get the Sequence Header Value for the Hub Numerical Identifier key from the Inverse Index Hash and append it to the value for the Cluster Numerical Identifier (from step 3.1 key in the Aggregate Cluster Hash.
  3.3. Append this Hub Numerical Identifier key - Cluster Numerical Identifier value pair to the Tracking Hash.
4. If neither the Hub nor Node have been previously assigned to a cluster in the Tracking Hash:
  4.1. Create an Aggregate Cluster Hash pair with a Cluster Numerical Identifier as the key and the sequence headers for the Hub and Node Numerical Identifiers from the Inverse Index Hash as the value. Append the Hub Numerical Identifier key - Cluster Numerical Identifier value to the Tracking Hash.
  4.2. Append the Node Numerical Identifier key - Cluster Numerical Identifier value to the Tracking Hash.
  4.3. Assign the next Cluster Numerical Identifier to be +1 greater than the current one (to create a new cluster).

This aggregating process is displayed as a flowchart in **Figure 1**.

## RESULTS

Each individual iteration of centroid-linkage clustering with randomized inputs should yield cluster distributions that are similar but not identical. Depending on the sequence input order, some sequences will not be clustered with the same matches for every iteration. Alternatively, some sequences will be so closely matched to other sequences that they will be grouped together in all or nearly all iterations. With enough iterations, the most prominent and closely-matched edges will appear more often than distant edges. Since these closely-matched sequences are likely to have edges that appear often, they will be among the first to be built into the aggregate clusters with the procedure outlined above.

**Figure 2.**
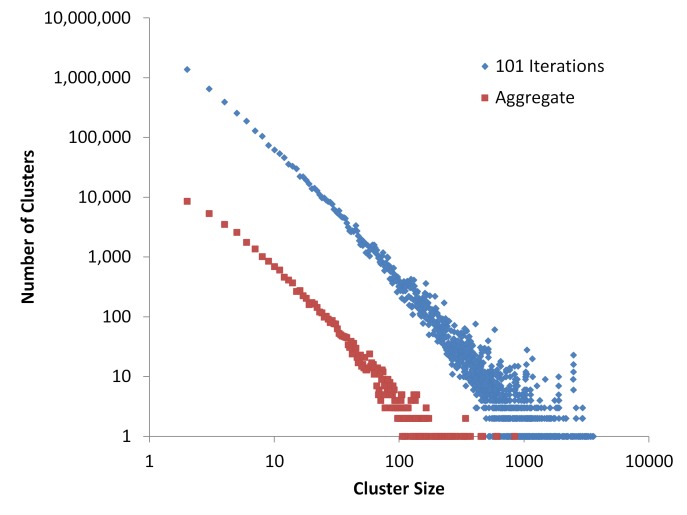
Cluster distributions of the individual iterations of centroid-linkage clustering (blue data points) and the aggregate clusters (red data points) for a dataset of one million sequences. Both axes are displayed in a logarithmic scale.

Aggregating the results of many iterations of centroid-linkage clustering builds clusters from high-consensus edges while cutting out low-consensus edges. The edges are ranked from highest to lowest consensus which is then followed in the aggregation process. This process generally results in the aggregate maximum cluster size being smaller than some clusters of the individual iterations, especially for larger sequence datasets, as seen in **Figure 2** for a dataset of one million sequences. The number of clusters produced by the aggregation process and a single iteration of centroid linkage clustering is shown in **Table 1** for multiple dataset sizes, which includes the data plotted in **Figure 2**. Sequences of low-consensus edges that are trimmed out by the aggregating process are either binned to clusters where they are part of a higher-consensus edge or they are binned as a single-sequence cluster. However, the two cluster distributions remain the same, as shown with Kolmogorov-Smirnov test in **Table 1.** Total runtime (which includes the 101 iteration of clustering) for this one millions sequence dataset was 120:36:56 (Hours:Minutes:Seconds). For datasets of other sizes: 5000 sequences, 00:43:11; 10000 sequences, 01:21:46; 50000 sequences, 01:50:33; 100000 sequences, 03:58:32; 500000 sequences, 54:44:34.

**Figure 3.**
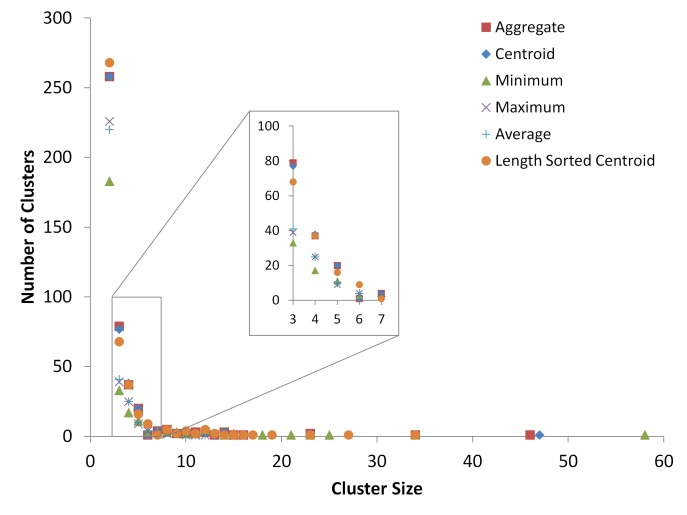
10000 sequences dataset cluster distributions for the aggregated clusters of Figure 1, as well as single clustering runs of centroid-, minimum-, maximum-, and average-linkage algorithms from USEARCH. The graph displays counts of all non-singleton clusters. The x-axis shows the size of the clusters produced from the five different methods, *i.e*., the number of sequences in each cluster. The y-axis shows the number of clusters that were produced of the sizes displayed on the x-axis.

The cluster distribution of the aggregate clusters follows the same pattern seen in the individual iterations, suggesting that the aggregation process does not drastically alter the cluster distributions of the centroid-linkage iterations to the point of being unrepresentative, as seen in **Figure 3.** In contrast, minimum-, maximum-, and average-linkage clustering algorithms yield a cluster distribution that varies more substantially from the centroid-linkage algorithm in **Figure 3.** **Table 2** shows Kolmogorov-Smirnov D statistics for pairwise comparisons between the cluster distributions shown in **Figure 3.** The table shows that the centroid method distribution’s least distant comparison is with the aggregate cluster distribution, with an estimated *P* value which does not allow us to reject the null hypothesis of having the same cluster distributions. This means that the aggregation process does reconstruct centroid-linkage cluster distribution instead of creating its own distinct cluster distribution. The data plotted in **Figure 3** is also displayed in tabular format in **Table 3**.

As mentioned in the introduction, pre-sorting sequences by length ensures that cluster centroids contain maximum information and thus cluster members can be binned more accurately. Conversely, abundance pre-sorting approaches accuracy with the assumption that abundant sequences are more likely to represent functionally relevant clusters. The aggregation process that we introduce clusters sequences with their most frequent edge counterpart from multiple iterations of random input-order centroid clustering. Our approach to accuracy is focused on the edges, using iterations of random input-order clustering to create a sorted, or ranked, edge list. Qualitatively, this has the effect of creating accurate clusters when presorting a sequence dataset by length/abundance is not sufficient or not possible.

As a simple example, a mock dataset of ten 100-base sequences populated *via* introducing one or zero random substitutions into a duplicate of the previous sequence was clustered using the aggregation process. In this dataset, listed below in FASTA format, with substitutions as capital letters, sequences mock0 and mock1 were identical, mock2 and mock3 were identical, and mock5 and mock6 were identical leading to a total of seven unique sequences. Sorting this mock sequence dataset by length or abundance does not yield a clear pre-sorted input. The aggregation process clusters mock0 and mock1 together and mock2–mock9 in a separate cluster. The edges between the sequences in these clusters occurred in 101/101 iterations of random input-order centroid clustering. Edges that connect the two clusters occurred in only 58/101 iterations, making them less of a priority in the aggregation algorithm. Length or abundance pre-sorting this mock dataset could yield either the single or double cluster distribution from the individual iterations depending on which sequence is chosen as the centroid sequence. Pre-sorting datasets with similar properties would yield clustering results that are close to a single random input-order iteration. Listed below are the mock DNA sequences described in the paragraph above.

>mock0

gaacaatgcattgtcattgctacaccgtttacatattacagagctttgcgcataagttcaacagcaccctggtcagctagagcacgatagcgcagcccct

>mock1

gaacaatgcattgtcattgctacaccgtttacatattacagagctttgcgcataagttcaacagcaccctggtcagctagagcacgatagcgcagcccct

>mock2

gaacaatgcattgtcatAgctacaccgtttacatattacagagctttgcgcataagttcaacagcaccctggtcagctagagcacgatagcgcagcccct

>mock3

gaacaatgcattgtcatAgctacaccgtttacatattacagagctttgcgcataagttcaacagcaccctggtcagctagagcacgatagcgcagcccct

>mock4

gaacaatgcattAtcatAgctacaccgtttacatattacagagctttgcgcataagttcaacagcaccctggtcagctagagcacgatagcgcagcccct

>mock5

gaacaatgcattAtcatAgctacaccgtttacatattacagagctttgcgcataagttcaacagcaccctggtGagctagagcacgatagcgcagcccct

>mock6

gaacaatgcattAtcatAgctacaccgtttacatattacagagctttgcgcataagttcaacagcaccctggtGagctagagcacgatagcgcagcccct

>mock7

gaacaatgcattAtcatAgctacaccgtttacatattacagagcCttgcgcataagttcaacagcaccctggtGagctagagcacgatagcgcagcccct

>mock8

gaacaatgcattAtcatAgctacacAgtttacatattacagagcCttgcgcataagttcaacagcaccctggtGagctagagcacgatagcgcagcccct

>mock9

gaacaatgcattAtcatAgctTcacAgtttacatattacagagcCttgcgcataagttcaacagcaccctggtGagctagagcacgatagcgcagcccct

## DISCUSSION

Since this aggregation process sacrifices speed to use less memory than agglomerative clustering while improving centroid-linkage clustering, the method can be much slower for large datasets. In addition, since data is written to disk storage instead of RAM, large datasets can require a large amount of available disk space, as mentioned in the final paragraph of the Introduction section. While the lengthier completion time and large amount of required disk space are drawbacks to this method, the aggregation process will eventually finish if these conditions are acceptable to the user.

**Figure 4.**
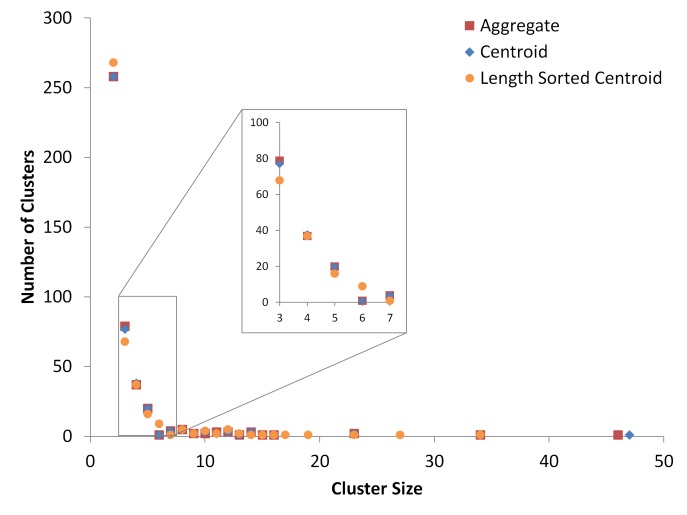
10000 sequences dataset cluster distributions for the aggregated clusters of Figure 1, as well as single clustering runs of centroid- and length sorted centroid-linkage algorithms from USEARCH. The graph displays counts of all non-singleton clusters. The x-axis shows the size of the clusters produced from the five different methods, *i.e*., the number of sequences in each cluster. The y-axis shows the number of clusters that were produced of the sizes displayed on the x-axis. All pairwise comparisons between results of the methods plotted in this figure had Kolmogorov-Smirnov *P* value of 1, meaning that the null hypothesis of the data having the same distribution cannot be rejected.

Alternative methods for improving centroid-clustering results include presorting the input sequences either by length, unique sequence abundance, or combination of the two [5,8]. **Figure 4** shows a comparison of the cluster distribution for the aggregated clusters, randomly sorted centroid-linkage, and length sorted centroid-linkage (sorted with the sort option in USEARCH). **Figure 4** **Table 4** (which shows the data in tabular format) show the cluster distribution from the aggregation process is closer to the distribution of the randomly sorted centroid-linkage than the length sorted, although not significantly so. However, both of these sorting methods (length and abundance) still produce results that are dependent on a single, and to some degree, arbitrary input order, while the aggregating process attempts to find the average result of many possible input orders. A possible middle ground would be to incorporate the results from presorted clustering to weight the aggregation inputs with as many iterations of presorted cluster distributions as desired. For example, if a user wanted to make sure that length sorted centroid-linkage was represented in the final aggregated cluster distribution, they could include length sorted results in place of one or more of the randomly sorted iterations. Unfortunately, just as between length and abundance sorted methods, it is difficult to say which method is definitively ‘better’ for most datasets.

In conclusion, aggregating randomly sorted centroid-linkage clustering results into a single distribution mitigates the consequences of input-order dependence in centroid-linkage clustering. The process described here primarily uses disk storage instead of RAM, which can have the consequences of long run times and requiring a large amount of available disk space. However, these consequences may be acceptable to researchers using a dataset that is too large for the distance matrices of agglomerative clustering methods. Centroid-linkage circumvents the need for constructing large distance matrices at the cost of input-order dependence. Methods exist to correct for this input-order dependence, such as presorting input sequences by length, unique sequence abundance, or combination of the two. While these methods may improve on the results of a single randomly sorted input order, they still represent a single, and to some degree, arbitrary input order. By aggregating the results of many randomly sorted iterations of centroid-linkage, the final result will not be dependent on any single input order. This method provides an alternative to the results from presorted centroid-linkage clustering.

## Supplementary Material

Supplementary information **File S1**. Annotated Perl script.
Supplementary information of this article can be found online at
http://www.jbmethods.org/jbm/rt/suppFiles/153.

## Figures and Tables

**Table 1.** Comparison of the number of non-singleton clusters between a single centroid-linkage iteration and the aggregate for datasets that range from 5000 to 1000000 sequences.

Dataset size (sequences)	Centroid Iteration	Aggregate	Kolmogorov-Smirnov *P* value
5000	172	174	1
10000	423	424	1
50000	1155	1212	1
100000	2693	2899	1
500000	17456	20728	0.4174
1000000	37487	311326	0.2468

The fourth column is Kolmogorov-Smirnov D statistic comparisons between centroid (single iteration) and aggregate cluster distributions for the six dataset sizes, as well as the data plotted **Figure 2.** The 1000000 sequences dataset had an estimated *P* value of 0.2468, the 500000 sequences dataset had an estimated *P* value of 0.4174, and all others had an estimated *P* value of 1, indicating for all datasets that the null hypothesis of the data having the same distribution cannot be rejected. Kolmogorov-Smirnov comparisons were performed in R with ks.test of the R Stats Package (r-project.org).

**Table 2.** Kolmogorov-Smirnov *P* value table for each pairwise comparison between results of the methods plotted in Figure 3.

	Centroid	Aggregate	Min.	Max.	Avg.
Centroid	1				
Aggregate	1	1			
Minimum	1	1	1		
Maximum	0.7833	0.7833	0.6284	1	
Average	0.9103	0.9103	0.7833	1	1

Kolmogorov-Smirnov calculations include singleton clusters, which are not plotted in **Figure 3.** No pairwise comparison estimated *P* value was smaller than 0.6284 (Minimum-Maximum comparison) meaning that the null hypothesis of the data having the same distribution cannot be rejected. Kolmogorov-Smirnov comparisons were performed in R with ks.test of the R Stats Package (r-project.org).

**Table 3.** Tabular format of the data plotted in Figure 3.

Cluster size	Agg.	Centroid	Min.	Max.	Avg.
1	8606	8608	8997	9167	9135
2	258	258	183	226	220
3	79	77	33	39	41
4	37	38	17	25	25
5	20	20	11	9	10
6	1	1	3	4	4
7	4	4	3	4	2
8	5	5	3	2	5
9	2	2	3	2	2
10	2	3	2	1	2
11	3	2	2	1	1
12	3	3	5	1	1
13	1	1	0	0	1
14	3	3	1	0	0
15	1	1	2	0	0
16	1	1	0	0	0
18	0	0	1	0	0
21	0	0	1	0	0
23	2	2	1	0	0
25	0	0	1	0	0
34	1	1	1	0	0
46	1	0	0	0	0
47	0	1	0	0	0
58	0	0	1	0	0

**Table 4.** Tabular format of the data plotted in Figure 4.

Cluster size	Aggregate	Centroid	Length sorted centroid
2	258	258	268
3	79	77	68
4	37	38	37
5	20	20	16
6	1	1	9
7	4	4	1
8	5	5	5
9	2	2	2
10	2	3	4
11	3	2	2
12	3	3	5
13	1	1	2
14	3	3	1
15	1	1	1
16	1	1	1
17	0	0	1
19	0	0	1
23	2	2	1
27	0	0	1
34	1	1	1
46	1	0	0
47	0	1	0
